# Associations of the composite dietary antioxidant index with all-cause mortality among individuals with psoriasis: a population-based study

**DOI:** 10.3389/fnut.2026.1594597

**Published:** 2026-04-14

**Authors:** Xuan Li, Yanqian Su, Jue Tang, Yanling He

**Affiliations:** Department of Dermatology, Beijing Chao-Yang Hospital, Capital Medical University, Beijing, China

**Keywords:** composite dietary antioxidant index, mortality, NHANES, oxidative stress, psoriasis

## Abstract

**Background:**

The composite dietary antioxidant index (CDAI) is an effective indicator to evaluate the comprehensive intake of dietary antioxidants in individuals. There is growing evidence suggesting that higher CDAI levels are associated with a reduced risk of diverse health conditions such as hypertension and atherosclerotic cardiovascular disease, as well as with lower mortality risk among the general public. However, its impact on the mortality risk of patients with psoriasis remains unclear. This study aimed to investigate the potential association between CDAI and the risk of death in individuals with psoriasis.

**Methods:**

Data extracted from the National Health and Nutrition Examination Survey (NHANES) spanning 2003–2006 and 2009–2014 was enrolled in this research, with mortality data obtained from the National Death Index (NDI) database. We utilized Multivariate COX proportional hazard regression models to investigate the influence of CDAI levels on the death risk from all causes among individuals with psoriasis. We further performed subgroup and sensitivity analyses to verify the reliability of the results.

**Results:**

Five hundred patients with psoriasis were finally enrolled in this prospective study. During the follow-up period, 61 deaths occurred. CDAI levels were linked to a reduced death risk in patients with psoriasis, compared to the lowest tertile, the hazard ratios for all-cause mortality were 0.48 (95% CI: 0.20–1.17, *P* = 0.105) for tertile 2 and 0.30 (95% CI: 0.12–0.74, *P* = 0.009) for tertile 3.

**Conclusion:**

Our study found that higher CDAI levels were associated with lower all-cause mortality in patients with psoriasis. Further prospective studies are needed to confirm the role of CDAI in mortality risk in patients with psoriasis.

## 1 Introduction

Psoriasis is a chronic and relapsing inflammatory skin condition that impacts roughly 125 million people all over the world, accounting for about 1%−3% of the global population (1). It has been regarded as a serious non-communicable disease by the World Health Organization. In companies with multiple serious medical conditions, including cardiovascular diseases (CVD), type 2 diabetes (T2D), psoriatic arthritis, psychological disorders, and inflammatory bowel disease, psoriasis can markedly reduce long-term quality of life and bring a substantial burden for not only individuals but also society (2–4). Previous studies have identified a significantly increased risk of mortality in patients with psoriasis (5).

It has been generally considered that the excessive production of reactive oxygen species (ROS) and the resulting oxidative stress are key factors in the development of psoriasis (6). Oxidative stress, stemming from an imbalance between prooxidants and antioxidants, is responsible for disrupting oxidation-reduction reactions (7). Previous studies have reported the redox imbalance in both skin and blood cells (such as granulocytes and lymphocytes) in psoriasis (8). Besides, mutations in the gene encoding Paraoxonase 1 (PON1), an enzyme with antioxidant function, have been found to be associated with the severity of psoriasis (9). Antioxidants can effectively scavenge free radicals, thereby reducing the inflammatory response to psoriasis and improving skin damage (10). Therefore, incorporating a diet rich in antioxidants has been suggested to be an effective non-pharmacologic intervention for psoriasis (8).

Single antioxidants do not accurately reflect an individual's overall antioxidant intake and fail to consider the synergistic effects between different antioxidant elements (11). The Composite Dietary Antioxidant Index (CDAI) serves as a practical indicator in evaluating the capacity of dietary antioxidants in individuals, by combining six essential antioxidant elements from the diet (vitamin A, vitamin C, vitamin E, zinc, selenium, and carotenoids) (12, 13). Previous research has provided evidence linking higher levels of CDAI to a reduced risk of depression, hypertension, and metabolic syndrome (14–16). However, little research links CDAI levels to the mortality risk among individuals with psoriasis.

Therefore, the latent connection between CDAI and mortality risk in psoriasis patients was investigated in this study.

## 2 Materials and methods

### 2.1 Data source

The National Health and Nutrition Examination Survey (NHANES) is a comprehensive cross-sectional survey conducted by the National Center for Health Statistics. It focuses on the health and nutritional status of civilians in the United States, and employs a stratified and multistage probability sampling method to choose participants, ensuring the representativeness of the general population. Data collection in NHANES is structured into five main sections: demographics, physical examinations, dietary assessments, laboratory tests, and questionnaire responses. These data are gathered by trained medical professionals to ensure accuracy and reliability. Additionally, NHANES includes follow-up mortality data, which is linked to the National Death Index (NDI), providing valuable information on participants' vital status over time. All protocols and procedures of NHANES undergo scrutiny and approval by the Ethics Review Board of the National Center for Health Statistics. Moreover, all participants are obligated to provide written informed consent before participating in the research, ensuring ethical compliance.

### 2.2 Study population

**Figure 1:**
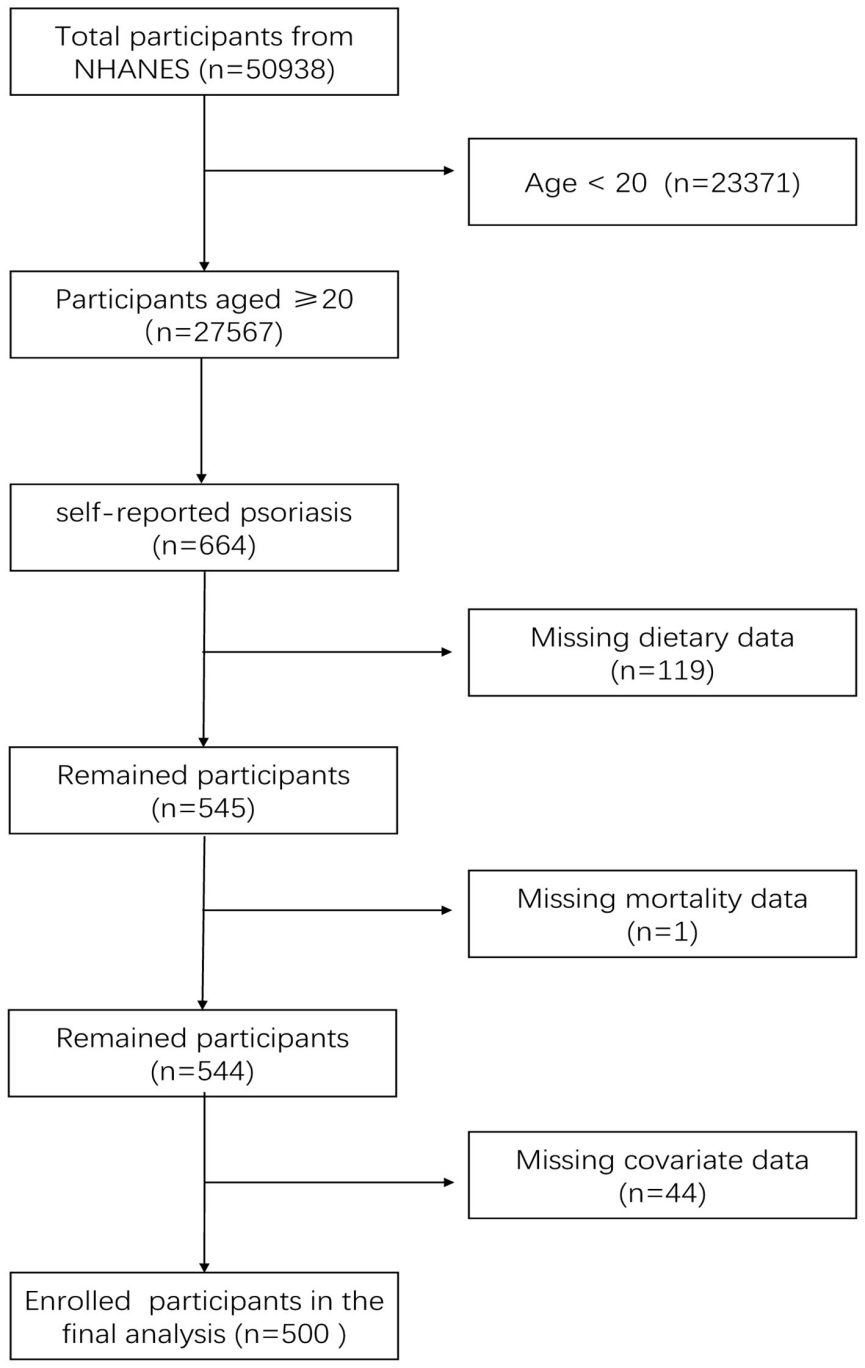
Flow chart of participant selection from NHANES 2003–2006 and 2009–2014.

A total of 50,938 individuals from five 2-year cycles (2003–2004, 2005–2006, 2009–2010, 2011–2012, 2013–2014) of NHANES were obtained in our study, Individuals (aged ≥20 years old) with psoriasis were specifically enrolled in this investigation (*n* = 664), and then excluded individuals with incomplete dietary intake data (*n* = 119), follow-up data (*n* = 1), and covariates with < 10% missing (*n* = 44), leaving 500 individuals with complete information in our final analysis (Figure 1). Any missing data less than 10% were regarded as random and were removed, while the multiple imputation technique was employed for variables with missing data ranging from more than 10% to less than 20%.

### 2.3 Assessment of composite dietary antioxidant index

A non-consecutive 2-day 24-h dietary recall interview by professional interviewers recorded each participant's nutrition intake. The first day of dietary recall was carried out in person, while the next daily dietary was recalled through telephone 3–10 days later. The daily intake of participants was averaged over 2 days of diet. This standardized methodology employs the 24-h recall to enhance accuracy and uses a spaced interval to capture day-to-day variation in diet, thereby providing a more reliable estimate of usual intake. CDAI was determined based on six dietary antioxidants, including vitamins A, C, and E, selenium, zinc, and carotenoids exclusively from food only, as proposed by Wright (12). Each antioxidant was standardized by subtracting the mean and dividing by the standard deviation (SD). The calculation formula is as described as follows:


$$\begin{array}{l} CDAI=\overset{6}{\underset{i=1}{\sum }}\frac{Individual\;Intake\;-Mean}{SD} \end{array}$$

### 2.4 Diagnosis of psoriasis

Data on psoriasis were derived from the questionnaire data, where the presence of psoriasis was determined based on a self-reported doctor diagnosis, and those who had been told they had psoriasis by a physician or other healthcare professional were defined as having psoriasis.

### 2.5 Ascertainment of mortality

Vital status was obtained from the NHANES-NDI linked file; NDI is a publicly accessible database (https://www.cdc.gov/nchs/data-linkage/mortality.htm) with accessible data through December 31, 2019. The causes of death for the participants were categorized using the International Classification of Diseases (ICD), with all-cause death encompassing any form of mortality listed within the classification.

### 2.6 Ascertainment of covariates

To diminish the potential impact of confounding factors, we conducted a literature search for studies on “CDAI” and “psoriasis.” By reviewing the “Statistical Analysis” or “Methods” sections of these publications, we extracted and summarized the covariates used for multivariable adjustment. Variables that were frequently reported and demonstrated clear biological or epidemiological relevance were identified as core candidates. Based on this, we screened the data from NHANES to include only variables with available data. Finally we identified several covariates including individual sex (male, female), age at interview, race (non-Hispanic white, other), education levels (high school or less, equivalent, college and above), family income to poverty ratio (PIR), matrimonial status (married/cohabiting, widowed/divorced/separated, never married), activity condition (active, inactive), cigarette smoking (non-smoker, smoker), alcohol consumption (non-drinker, drinker), and body mass index (BMI; < 25, 25–29.9, ≥30 kg/m^2^). Non-smokers were classified as participants who had smoked less than 100 cigarettes over their lifetime, whereas those who had smoked more than 100 cigarettes were categorized as smokers. Alcohol drinking status was ascertained by the answer to the question “Have you/she/he had at least 12 drinks of any type of alcoholic beverage in your/her/his entire life?” Physical activity level was divided into two groups, those who engage in moderate to vigorous-intensity exercise, fitness plans, or recreational activities for more than 10 min per week were defined as active, while those who exercise for less than 10 min per week were defined as inactive.

### 2.7 Statistical analysis

In analyses utilizing the NHANES database, factors such as sample weights, clustering, and stratification, which are part of the complex survey design, were duly considered. Initially, participants were segregated into two groups according to their survival status at the follow-up cut-off date, and comparisons of baseline characteristics were conducted by Student's *t*-tests for continuous variables and chi-square tests for categorical variables. For categorical variables (sex, race, education level, matrimonial status, BMI, cigarette smoking, alcohol consumption, activity condition, and CDAI tertiles), counts and proportions were presented, while means and standard deviation were presented for continuous variables (age, PIR, and CDAI).

Restricted cubic spline analysis (RCS) was employed to explore the nonlinear relationship between CDAI levels and all-cause mortality. Subsequently, a Kaplan-Meier analysis was conducted to assess the difference in survival among three CDAI groups of psoriasis patients, followed by significance testing using the Log-rank test.

Subsequently, we applied multivariate Cox proportional regression models to assess the risk proportions (HRs) and 95% confidence intervals (CIs) regarding the relationship of CDAI (with CDAI tertile 1 as reference) and all-cause death risk in psoriasis patients. Firstly, model 1 is a crude model without any covariate adjustments. Then, in model 2, adjustments were made for sex, age, and race. Finally, in model 3, we further adjusted PIR, education level, matrimonial status, cigarette smoking, alcohol consumption, activity condition, and BMI. In addition to Cox regression, we performed trend tests for the tertile groups of CDAI.

Moreover, several sensitivity analyses were carried out to verify the robustness of these findings and address potential reverse causation bias. Firstly, individuals who passed away within the first 6 months from the baseline were eliminated, followed by a re-evaluation of Cox proportional regression in model 3. Secondly, adjustments were made for participants with or without a history of metabolic syndrome, congestive heart failure (CHF), diabetes, CVD, stroke, and cancer at the baseline for analysis. Furthermore, stratified analyses were conducted to assess whether the results varied by age group (20–59 or ≥60 years old), sex (male or female), race and ethnicity (non-Hispanic white or other), BMI (< 30 or ≥30 kg/m^2^), cigarette smoking (non-smoker or smoker), physical activity condition (inactive or active).

R 4.3.1 was utilized for all statistical analyses, with a significance level set at a *P*-value less than 0.05 for all tests conducted in this study.

## 3 Results

### 3.1 Baseline characteristics

There are 500 participants (mean age, 47.43 ± 15.05 years; 234 male and 266 female) in this study. During the follow-up duration, 61 deaths occurred. Table 1 describes the weighted features of the baseline psoriasis patients classified by survival status. In this study, the CDAI, which represents the antioxidant capacity, was prominently lower in the group of deceased individuals compared to the surviving group (−1.69 and 0.15, respectively; *P* = 0.003). Common factors such as education levels, PIR, matrimonial status, BMI, cigarette smoking, physical activity condition, and CDAI tertiles differed significantly between the two groups. But there is no difference noted in terms of sex, race, and alcohol consumption.

**Table 1:** Baseline characteristics of adults with psoriasis classified by survival status.

Characteristic	Total (*N* = 500)	Survival status		*P*-value
		Alive (*n* = 439)	Deceased (*n* = 61)	
Age, years	47.43 (15.05)	46.02 (14.41)	62.90 (13.27)	< 0.001
Sex				0.089
Male	234.00 (52.64%)	204.00 (53.77%)	30.00 (40.19%)	
Female	266.00 (47.36%)	235.00 (46.23%)	31.00 (59.81%)	
Race and ethnicity				0.088
Non-Hispanic white	312.00 (80.32%)	264.00 (79.64%)	48.00 (87.76%)	
Other	188.00 (19.68%)	175.00 (20.36%)	13.00 (12.24%)	
Education level				0.026
High school or less	206.00 (33.45%)	173.00 (31.82%)	33.00 (51.37%)	
Equivalent	159.00 (34.14%)	138.00 (34.07%)	21.00 (34.80%)	
College and above	135.00 (32.41%)	128.00 (34.11%)	7.00 (13.83%)	
Family income to poverty ratio (PIR)	3.22 (1.68)	3.29 (1.67)	2.49 (1.70)	0.020
Matrimonial status				0.047
Married/cohabiting	305.00 (68.31%)	273.00 (69.73%)	32.00 (52.74%)	
Widowed/divorced/separated	120.00 (16.39%)	95.00 (14.74%)	25.00 (34.49%)	
Never married	75.00 (15.30%)	71.00 (15.53%)	4.00 (12.77%)	
**BMI, kg/m** ^2^				0.004
< 25.0	115.00 (25.97%)	106.00 (26.95%)	9.00 (15.27%)	
25.0–29.9	164.00 (35.63%)	147.00 (36.91%)	17.00 (21.57%)	
≥30.0	221.00 (38.40%)	186.00 (36.14%)	35.00 (63.16%)	
Cigarette smoking				0.037
Non-smoker	219.00 (41.74%)	206.00 (43.39%)	13.00 (23.66%)	
Smoker	281.00 (58.26%)	233.00 (56.61%)	48.00 (76.34%)	
Alcohol consumption				0.980
Non-drinker	59.00 (8.20%)	53.00 (8.19%)	6.00 (8.29%)	
Drinker	441.00 (91.80%)	386.00 (91.81%)	55.00 (91.71%)	
Activity condition				0.017
Inactive	230.00 (42.11%)	191.00 (40.28%)	39.00 (62.27%)	
Active	270.00 (57.89%)	248.00 (59.72%)	22.00 (37.73%)	
CDAI	0.00 (4.01)	0.15 (3.97)	−1.69 (4.08)	0.003
CDAI tertiles				< 0.001
Tertile 1	214.00 (33.07%)	177.00 (30.43%)	37.00 (62.07%)	
Tertile 2	153.00 (33.43%)	140.00 (34.42%)	13.00 (22.54%)	
Tertile 3	133.00 (33.50%)	122.00 (35.15%)	11.00 (15.39%)	

All estimates incorporate sampling weights and adjust for the complex, multistage sampling design of NHANES to produce nationally representative results. The baseline characteristics of participants were conducted by Student's t-tests for continuous variables and chi-square tests for categorical variables. For categorical variables (sex, race, education level, matrimonial status, BMI, cigarette smoking, alcohol consumption, activity condition, and CDAI tertiles), counts and proportions were presented, while means and standard deviation were presented for continuous variables (age, PIR, and CDAI).

### 3.2 The relationship between CDAI and mortality risk in adults with psoriasis

**Figure 2:**
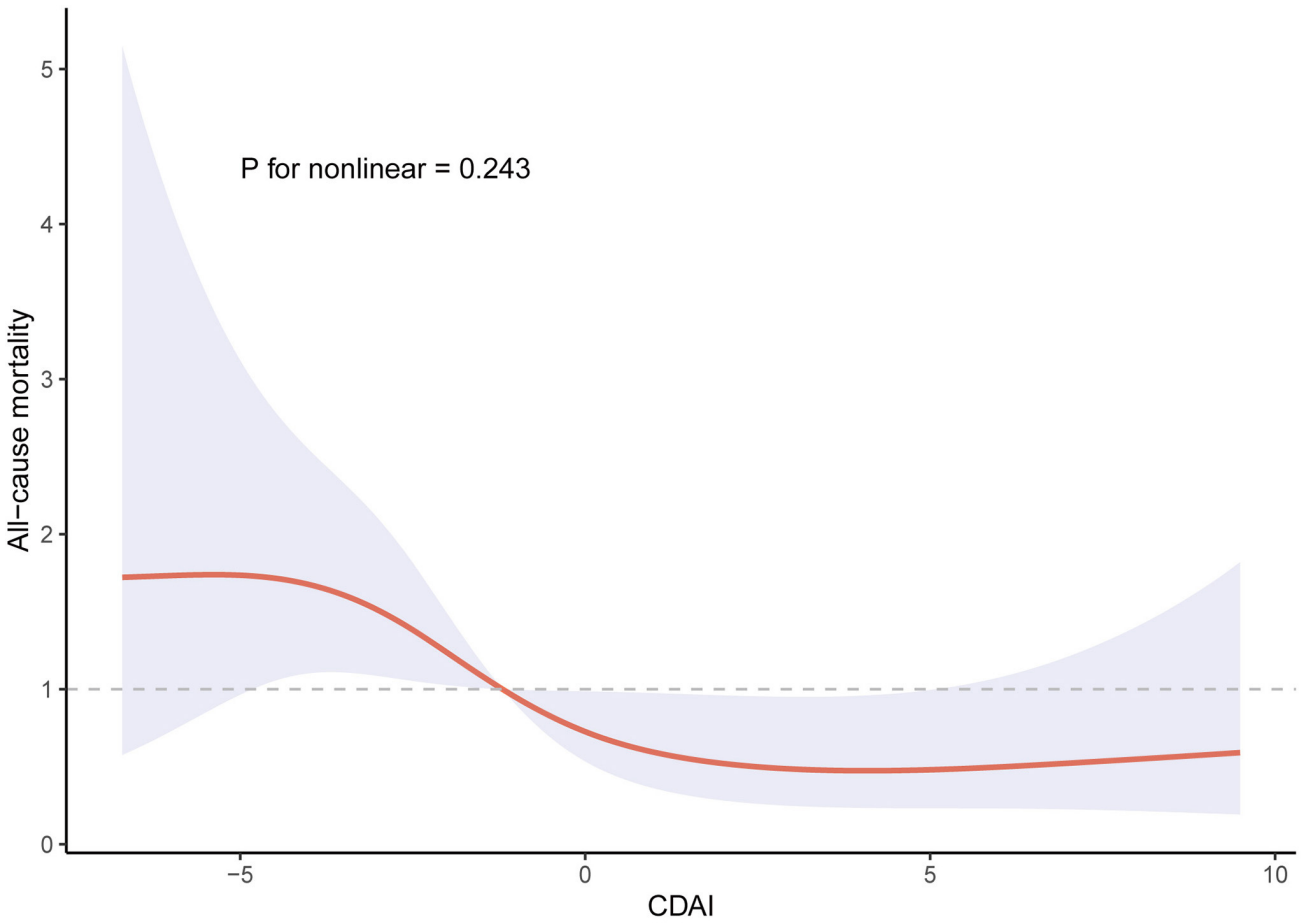
Restricted cubic splines for the association between CDAI and all-cause mortality in individuals with psoriasis.

**Figure 3:**
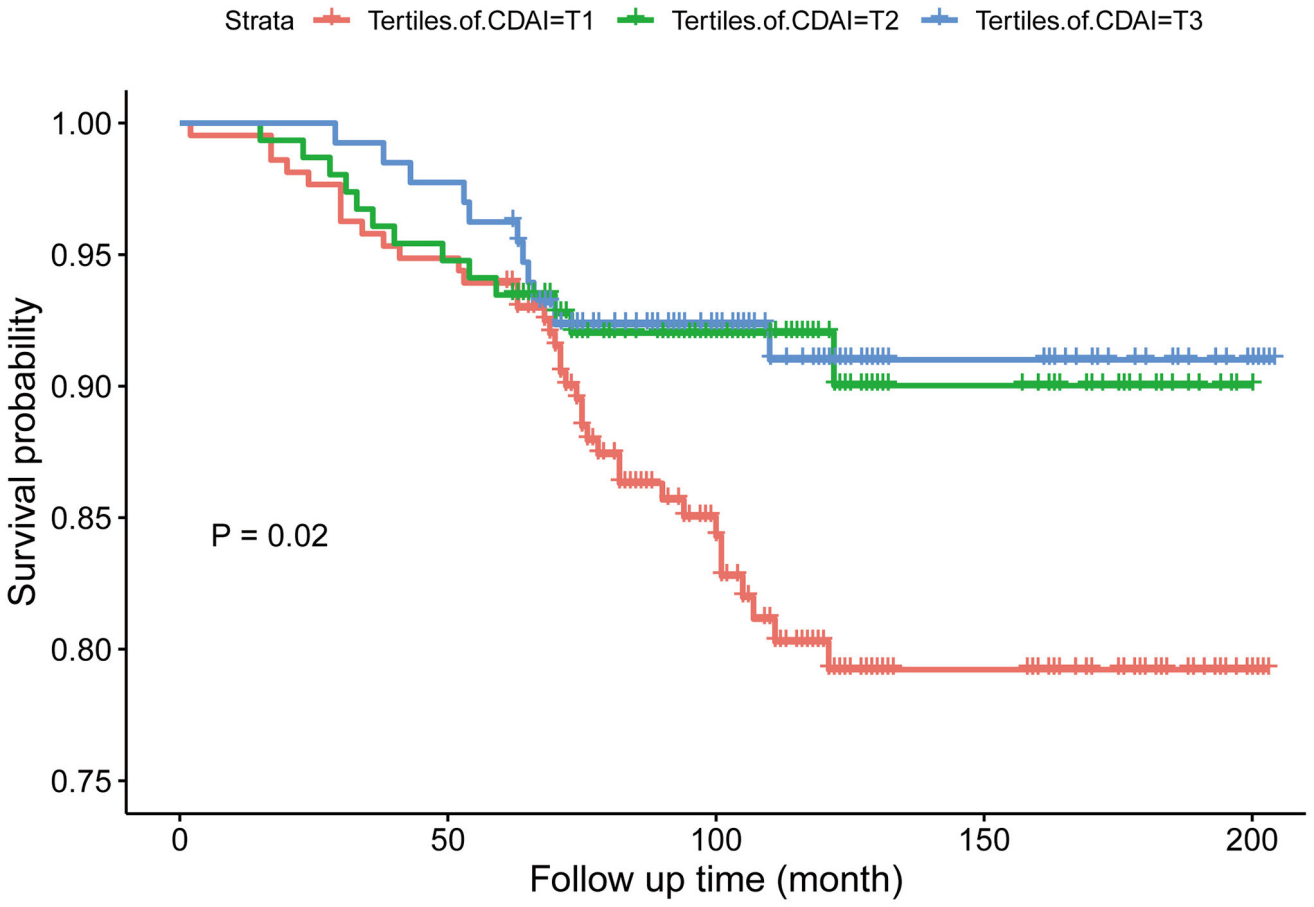
Kaplan-Meier survival curves for all-cause mortality among individuals with psoriasis, stratified by tertiles of the CDAI.

The RCS analysis depicted in Figure 2 did not detect a significant nonlinear association between CDAI and the risk of death (*P* for nonlinear = 0.243). As shown in Figure 3, the Kaplan-Meier analysis suggested that a higher level of CDAI was related to a lower incidence of all-cause mortality in psoriasis patients (Log-rank test *P* = 0.02).

As described in Table 2, elevated CDAI levels were associated with a lower risk of all-cause mortality in psoriasis adults across progressively adjusted models. Stratifying CDAI tertiles into three categories (T1 [−7.95, −1.87], T2 (−1.87, 1.09], T3 (1.09, 39.58]), it was observed that T3 (HR: 0.30, 95% CI: 0.12–0.74, *P* = 0.009) was linked to a significantly reduced risk of all-cause mortality in the multivariable-adjusted model (model 3) compared to the reference tertile (T1), and T2 (HR: 0.48, 95% CI: 0.20–1.17, *P* = 0.105). The *P*-value for the trend test was 0.005 after adjusting for these covariates.

**Table 2:** Association of the Composite Dietary Antioxidant Index with all-cause mortality in individuals with psoriasis.

Variables	Model 1		Model 2		Model 3	
All-cause mortality	HR (95% CI)	*P*	HR (95% CI)	*P*	HR (95% CI)	*P*
CDAI	0.88 (0.78, 0.99)	**0.029**	0.88 (0.78, 0.99)	**0.038**	0.9 (0.81, 1.00)	0.062
Classification						
Tertile 1	Ref	Ref	Ref	Ref	Ref	Ref
Tertile 2	0.39 (0.18, 0.85)	**0.018**	0.41 (0.18, 0.96)	**0.040**	0.48 (0.20, 1.17)	0.105
Tertile 3	0.24 (0.09, 0.60)	**0.003**	0.29 (0.12, 0.70)	**0.006**	0.30 (0.12, 0.74)	**0.009**
*P* for trend		**0.003**		**0.006**		**0.005**

Model 1 was adjusted for none. Model 2 was adjusted for age, sex, and race. Model 3 was adjusted for age, sex, race, family income to poverty ratio, education level, matrimonial status, cigarette smoking, alcohol consumption, activity condition, and BMI. Bold values indicates *P* < 0.05.

### 3.3 Subgroup and sensitivity analysis

**Figure 4:**
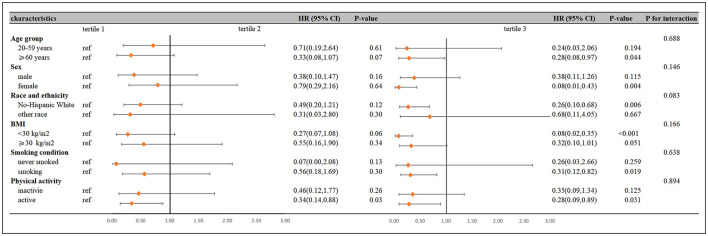
Subgroup analysis of the association between CDAI and all-cause mortality in individuals with psoriasis.

Subgroup analyses stratified by concomitant variables showed that the protective effect of high CDAI levels on mortality remains statistically significant, and no remarkable interactions were detected between CDAI levels with these subgroup analysis factors (all *P* for interaction >0.05; Figure 4).

Besides, excluding individuals who passed away within the first 6 months following the baseline interview didn't weaken the inverse correlation between CDAI and death risk (HR: 0.32, 95% CI: 0.13–0.80, *P* = 0.014; Table 3). And the significant association persisted after further adjusting the history of metabolic syndrome, CHF, diabetes, CVD, stroke, and cancer at baseline for tertile 3 in model 3 (HR: 0.35, 95% CI: 0.13–0.96, *P* = 0.041). Moreover, to examine whether the observed association between CDAI and all-cause mortality was independent of major comorbidities, we restricted the analysis to a cohort of 280 psoriasis patients without common comorbidities at baseline, specifically excluding those with reported cardiovascular disease, diabetes, metabolic syndrome, or cancer. In this cohort, the inverse association between higher CDAI levels and lower mortality risk persisted, with results consistent with the primary analysis (HR: 0.06, 95% CI: 0.00–0.70, *P* = 0.025; Supplementary Table S1).

**Table 3:** Association of the Composite Dietary Antioxidant Index with all-cause mortality in individuals with psoriasis after excluding deaths within the first 6 months.

Variables	Model 1		Model 2		Model 3	
All-cause mortality	HR (95% CI)	*P*	HR (95% CI)	*P*	HR (95% CI)	*P*
CDAI	0.88(0.78, 0.99)	**0.029**	0.88 (0.78, 0.99)	**0.038**	0.9 (0.81, 1.00)	0.062
Classification						
Tertile 1	Ref	Ref	Ref	Ref	Ref	Ref
Tertile 2	0.39 (0.18, 0.87)	**0.020**	0.42 (0.18, 0.98)	**0.046**	0.50 (0.21, 1.21)	0.120
Tertile 3	0.24 (0.09, 0.62)	**0.003**	0.29 (0.12, 0.72)	**0.008**	0.32 (0.13, 0.80)	**0.014**
*P* for trend		**0.003**		**0.008**		**0.009**

Model 1 was adjusted for none. Model 2 was adjusted for age, sex, and race. Model 3 was adjusted for age, sex, race, family income to poverty ratio, education level, matrimonial status, cigarette smoking, alcohol consumption, activity condition, and BMI. Bold values indicates *P* < 0.05.

The exploration of the correlation between components of CDAI and risk of death was added to the sensitive analysis, which suggested that only vitamin E (HR: 0.34, 95% CI: 0.13–0.91, *P* = 0.031) and zinc (HR: 0.24, 95% CI: 0.10–0.57, *P* = 0.001) were independently correlated to the risk of death in psoriasis individuals after adjusting multiple covariates (Table 4).

**Table 4:** Association of individual components of the Composite Dietary Antioxidant Index with all-cause mortality in adults with psoriasis.

Variables	Model 1		Model 2		Model 3	
All-cause mortality	HR (95% CI)	*P*	HR (95% CI)	*P*	HR (95% CI)	*P*
Vitamin A						
Tertile 1	Ref	Ref	Ref	Ref	Ref	Ref
Tertile 2	0.71 (0.36, 1.40)	0.324	0.52 (0.26, 1.04)	0.065	0.73 (0.38, 1.41)	0.353
Tertile 3	0.67 (0.27, 1.65)	0.383	0.58 (0.20, 1.70)	0.325	0.59 (0.18, 1.95)	0.390
Vitamin C						
Tertile 1	Ref	Ref	Ref	Ref	Ref	Ref
Tertile 2	0.59 (0.25, 1.37)	0.22	0.48 (0.22, 1.07)	0.072	0.56 (0.24, 1.28)	0.166
Tertile 3	0.57 (0.24, 1.33)	0.191	0.44 (0.20, 0.94)	**0.034**	0.48 (0.22, 1.03)	0.059
Vitamin E						
Tertile 1	Ref	Ref	Ref	Ref	Ref	Ref
Tertile 2	0.97 (0.47, 1.97)	0.927	0.79 (0.40, 1.58)	0.513	0.86 (0.45, 1.62)	0.632
Tertile 3	0.33 (0.13, 0.84)	**0.020**	0.37 (0.16, 0.88)	**0.024**	0.34 (0.13, 0.91)	**0.031**
Zinc						
Tertile 1	Ref	Ref	Ref	Ref	Ref	Ref
Tertile 2	0.28 (0.12, 0.63)	**0.002**	0.27 (0.13, 0.59)	**0.001**	0.38 (0.19, 0.75)	**0.005**
Tertile 3	0.16 (0.07, 0.39)	**< 0.001**	0.23 (0.09, 0.60)	**0.002**	0.24 (0.10, 0.57)	**0.001**
Selenium						
Tertile 1	Ref	Ref	Ref	Ref	Ref	Ref
Tertile 2	0.76 (0.36, 1.62)	0.447	0.8 (0.35, 1.82)	0.597	0.90 (0.36, 2.25)	0.829
Tertile 3	0.62 (0.27, 1.44)	0.266	0.87 (0.32, 2.35)	0.777	0.86 (0.28, 2.61)	0.791
Carotenoids						
Tertile 1	Ref	Ref	Ref	Ref	Ref	Ref
Tertile 2	0.44 (0.23, 0.84)	**0.012**	0.45 (0.22, 0.90)	**0.023**	0.60 (0.31, 1.18)	0.140
Tertile 3	0.43 (0.15, 1.18)	0.102	0.43 (0.16, 1.11)	0.08	0.44 (0.15, 1.29)	0.134

Model 1 was adjusted for none. Model 2 was adjusted for age, sex, and race. Model 3 was adjusted for age, sex, race, family income to poverty ratio, education level, matrimonial status, cigarette smoking, alcohol consumption, activity condition, and BMI. Bold values indicates *P* < 0.05.

## 4 Discussion

As we know, this study represents the first investigation focusing on patients with psoriasis and examining the impact of dietary antioxidant intake on the death risk in this specific population. This nationally representative study conducted in America unveiled a clear negative linear correlation between elevated levels of CDAI and all-cause death risk in psoriasis patients. Even upon adjusting for various confounders, this inverse correlation remains statistically significant. Furthermore, additional subgroup and sensitivity analyses corroborated the robustness of our findings, with subgroup analyses revealing that elderly individuals, females, smokers, those with high BMI, and regular exercisers tend to derive greater benefits from elevated CDAI levels.

CDAI has emerged as a widely employed metric in antioxidant-related investigations, serving to quantify an individual's antioxidant capacity. Previous research by Wang and Yi (17) unveiled a negative correlation between CDAI levels and all-cause mortality as well as cardiovascular mortality in the general public. This correlation extended to individuals with diabetes, stroke, and tumors as well (18–20). Nonetheless, conflicting outcomes have arisen in certain studies. For instance, one study found no mortality impact from dietary antioxidants among elderly individuals with heightened cardiovascular disease risk (21). Besides, another study did not observe a reduction in cardiovascular or all-cause mortality with antioxidant supplementation and reported a potential association with increased risk (22). Despite this, the existing evidence on CDAI and the long-term health prognosis among adults with psoriasis remains limited. Our current study contributes to this field by demonstrating an association between higher CDAI levels and reduced all-cause mortality, suggesting that higher dietary antioxidant intake may be associated with an improved long-term prognosis in patients with psoriasis.

The precise mechanisms underlying how CDAI affects the mortality of psoriasis patients are still elusive. One plausible explanation is that CDAI influences oxidative stress in psoriasis, thereby impacting the progression of inflammation in the condition. Oxidative stress denotes an imbalance in oxidation-reduction processes triggered by increased production of ROS and reactive nitrogen species (RNS), alongside a decline in antioxidant activity or concentration. In the context of psoriasis, a state of systemic and cutaneous redox imbalance is well-established. Evidence indicates significantly elevated levels of oxidative stress markers, such as malondialdehyde (MDA) and lipid hydroperoxides, in the lesional skin, plasma, and serum of psoriatic patients, accompanied by a decline in antioxidant defenses and decreased activity of key antioxidant enzymes, including superoxide dismutase (SOD) and catalase (23, 24). Given that the skin is consistently subjected to unfavorable external elements, it serves as a pivotal source of free radicals. Oxidative stress resulting from elevated levels of free radicals leads to DNA alteration, degradation of cellular proteins, lipid oxidation, cell apoptosis, and tissue damage (25, 26).

Mechanistically, oxidative stress is not merely a bystander but an active driver in the pathogenesis of psoriasis. Signaling pathways such as NF-κB, MAPK/AP1 and JAK-STAT play crucial roles in the pathogenesis of psoriasis, and ROS can modulate these signaling pathways, thereby activating immune cells, particularly Th-1 and Th-17 cells (27, 28). Consequently, this activation enhances the expression of pro-inflammatory chemokines and cytokines that ultimately contribute to the hyperproliferation of keratinocytes, infiltration of immune cells into the skin, and lipid peroxidation affecting vascular permeability, all of which are critical features of psoriasis (23, 29). A reinforcing loop was revealed recently, where the psoriatic cytokine cocktail M5 can provoke ROS generation, in turn, activates kinase cascades such as JNK/c-Jun, which further amplifies the activity of NF-κB, establishing a self-perpetuating inflammatory circuit (30). Notably, keratinocyte-derived ROS act as potent chemoattractants for neutrophils, whose subsequent recruitment and activation release further bursts of ROS, creating a chain reaction that exacerbates local oxidative stress and chronic inflammation (31). Additionally, aging is associated with elevated ROS levels, giving rise to oxidative stress, thus activating systemic pro-inflammatory pathways and accelerating the progression of psoriasis (32). These factors may elucidate why subgroup analysis highlighted a more pronounced impact of CDAI on mortality rates among the elderly population.

It is plausible that the association between a higher CDAI and improved survival could be partially explained by a concurrent association with a reduced risk of death from comorbid conditions. Individuals with psoriasis tend to have a higher prevalence of cardiovascular disease and type 2 diabetes, and existing evidence suggests that dietary antioxidant intake is associated with a lower risk of mortality from these specific conditions (18, 33). Dietary antioxidants can act as external factors that regulate plasma redox status, safeguarding the body against detrimental effects caused by ROS and RNS, thereby diminishing oxidative stress, alleviating systemic inflammation, and further lowering the mortality risk associated with psoriasis and its complications (34).

There are several notable strengths to this study. Firstly, the samples were carefully selected from a nationally representative database, making the conclusions convincing and allowing for extrapolation to the broader population of psoriasis at the national level. Secondly, rather than relying on a single antioxidant element, this study assessed the dietary antioxidant intake of individuals by calculating the CDAI, thereby minimizing potential confounding effects arising from interactions between different antioxidant elements and bolstering the reliability of the findings. Finally, rigorous subgroup and sensitivity analyses were conducted to eliminate the impact of potential confounding factors and demonstrate the reliability of the results.

Nevertheless, the limitations of the study must also be acknowledged. First, the identification of self-reported psoriasis was based on a single question, potentially overlooking individuals who may have been affected by psoriasis. Second, since this study was conducted exclusively in the United States, the results should be generalized with caution to populations in other countries. Third, the assessment of antioxidant intake relied on a 24-h dietary recall interview, which may be susceptible to recall bias, and the dietary habits may change over the long term, but we have no way of knowing due to limited information availability. In addition, it is worth noting that no data regarding the severity of psoriasis was included, thus individualization for patients with varying levels of severity could not be achieved. Finally, despite our efforts to adjust for several well-known confounding variables, there are still numerous potential factors that could potentially influence the outcomes. Moreover, in the subgroup analysis of patients without common comorbidities (*n* = 280), the relatively small sample size limits the statistical power and precision of the estimate. Beyond these methodological considerations, we were unable to directly validate this hypothesis in our study due to the lack of measurement data on oxidative stress biomarkers in NHANES. This confines the conclusions of this study to the level of epidemiological association. Future research specifically designed to prospectively collect detailed dietary data alongside a panel of oxidative stress biomarkers is needed to provide more direct evidence concerning the underlying causal mechanisms.

## 5 Conclusion

In this nationally representative investigation, we found that increased CDAI corresponded with a reduced all-cause death risk among psoriasis patients in the United States. These observational findings suggest that diets rich in antioxidants may hold prognostic significance in patients with psoriasis, which underscores the association between dietary patterns and long-term health outcomes in this population. Prospective studies, particularly interventional trials, are needed to confirm this association and explore potential causal mechanisms.

## Data Availability

The original contributions presented in the study are included in the article/Supplementary material, further inquiries can be directed to the corresponding author.
