# Supplementary material for: Associations of the composite dietary antioxidant index with all-cause mortality among individuals with psoriasis: a population-based study
Source: Front Nutr. 2026 Apr 14;13:1594597. doi: 10.3389/fnut.2026.1594597 (PMC13120939; doi:10.3389/fnut.2026.1594597)
Supplement: Supplementary file 1 [file Table_1.docx]

Supplementary Table S1. Relationship between CDAI and mortality in psoriasis patients without common comorbidities.

| **Variables** | **Model 1** |  | **Model 2** |  | **Model 3** |  |
| --- | --- | --- | --- | --- | --- | --- |
| All-cause mortality | HR (95% CI) | P | HR (95% CI) | P | HR (95% CI) | P |
| CDAI | 0.76(0.66,0.88) | **<0.001** | 0.78 (0.66,0.92) | **0.003** | 0.77 (0.63,0.94) | **0.010** |
| Classification |  |  |  |  |  |  |
| Tertile 1 | ref | ref | ref | ref | ref | ref |
| Tertile 2 | 0.35 (0.09,1.41) | 0.14 | 0.41 (0.13,1.30) | 0.13 | 0.33 (0.10,1.13) | 0.077 |
| Tertile 3 | 0.05 (0.01,0.40) | **0.005** | 0.06 (0.01,0.54) | **0.012** | 0.06 (0.00,0.70) | **0.025** |
| P for trend |  | **0.005** |  | **0.012** |  | **0.037** |

Model 1 was adjusted for none. Model 2 was adjusted for age, sex and race. Model 3 was adjusted for age, sex, race, Family income to poverty ratio, education level, matrimonial status, cigarette smoking, alcohol consumption, activity condition, and BMI.
